# Anorectal Malformations Associated With Labioscrotal Fold Malformation and Perineal Mass in Pediatric Patients: Over a Decade of Experience

**DOI:** 10.3389/fped.2021.627188

**Published:** 2021-02-10

**Authors:** Kai Wang, Chunhui Peng, Wenbo Pang, Zengmeng Wang, Dongyang Wu, Dan Zhang, Sarah Tan Siyin, Yajun Chen

**Affiliations:** Department of General Surgery, National Center for Children's Health, Beijing Children's Hospital, Capital Medical University, Beijing, China

**Keywords:** anorectal malformation, labioscrotal fold malformation, perineal mass, pediatric, treatment

## Abstract

**Background:** The triad of anorectal malformation (ARM), labioscrotal fold malformation, and perineal mass has rarely been reported before. The purpose of this study was to review our experience in these patients, describe their characteristics, and discuss the possible pathogenesis.

**Methods:** Seven pediatric patients diagnosed with ARM associated with both labioscrotal fold malformation and perineal mass were included in this study. Medical records of these patients were retrospectively reviewed, and follow-up was held through telephone contact or outpatient service.

**Results:** Among the seven patients were six females and one male, and the age at surgery was between 5.2 and 12.4 months. The ratio of lateral-type to mid-perineum-type labioscrotal fold malformation was 5:2. The ARM type was all rectoperineal fistula. Operation was excision of the malformation and perineal mass at the same time of anoplasty. The pathology was lipoma (three cases), fibroma (one case), lipofibroma (one case), angiolipoma (one case), and mesenchymal hamartoma (one case). All the seven patients had no wound complication, and during the follow-up period of 7–100 months after surgery, none of the seven patients suffered perineal mass recurrence. Bowel control was satisfactory in the follow-up period.

**Conclusions:** There is a low incidence for the triad of ARM, labioscrotal fold malformation, and perineal mass. The nature of this disease is neoplastic overgrowth of intervening mesenchymal tissue, which impedes the continuity of caudal development into normal labioscrotal fold and affects the extension of urorectal septum, leading to ARM. Prognosis is mainly dependent on the type of ARM.

## Introduction

Anorectal malformation (ARM) has an estimated incidence of 1 in 4,000–5,000 live births (1). Accompanying malformations, which play an important role in the comprehensive assessment before surgical treatment, usually complicate the treatment and can sometimes worsen the prognosis. Apart from the common anomalies that involve cardiac, spinal, urogenital, and digestive systems, labioscrotal fold malformation and perineal mass are rarely encountered and seldom described in ARM patients. A few cases reviewing independent labioscrotal fold malformation or perineal lipoma without ARM have been reported (2, 3). However, there is a particular group of ARM patients who have both labioscrotal fold malformation and perineal mass. Characteristics and pathogenesis of such patients have yet to be discussed and revealed.

The purpose of this study was to retrospectively review our experience in treating ARM patients with both labioscrotal fold malformation and perineal mass over the past 10 years, describe their characteristics, and discuss the possible pathogenesis for better understanding.

## Materials and Methods

ARM pediatric patients who were diagnosed with both labioscrotal fold malformation and perineal mass and who underwent surgery between January 2010 and July 2020 in the General Surgery Department of our hospital were included in this study. The medical records of patients were reviewed retrospectively. The following information was extracted for analysis: gender, age at surgery, type of ARM, site of labioscrotal fold malformation and perineal mass, associated anomalies, surgical procedures, and pathology of the mass. Follow-up sessions were held through telephone contact or outpatient service to understand the post-operative local condition and fecal continence.

## Results

**Figure 1:**
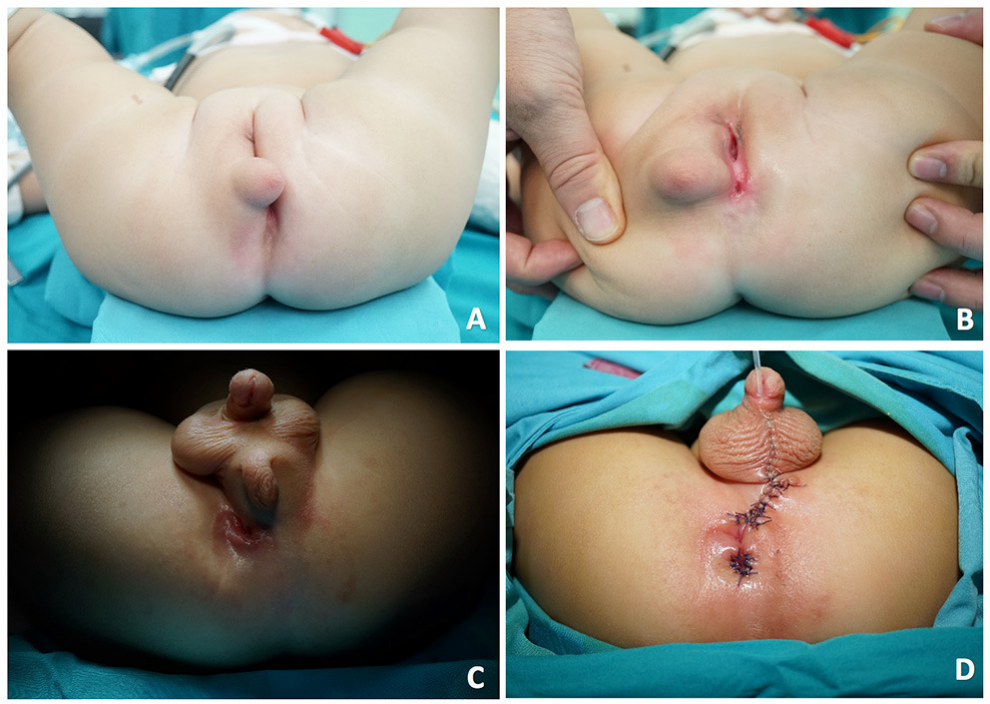
Appearance of the accessory labioscrotal fold on the perineal mass. **(A,B)** showed such a female patient (case 6) with rectoperineal fistula, and the perineal mass was on the right labium major. **(C)** showed a hypospadia, rectoperineal fistula male patient (case 7) with a perineal mass close to the scrotum and an accessory labioscrotal fold on the mass. **(D)** was the post-operative view of the case 7.

**Figure 2:**
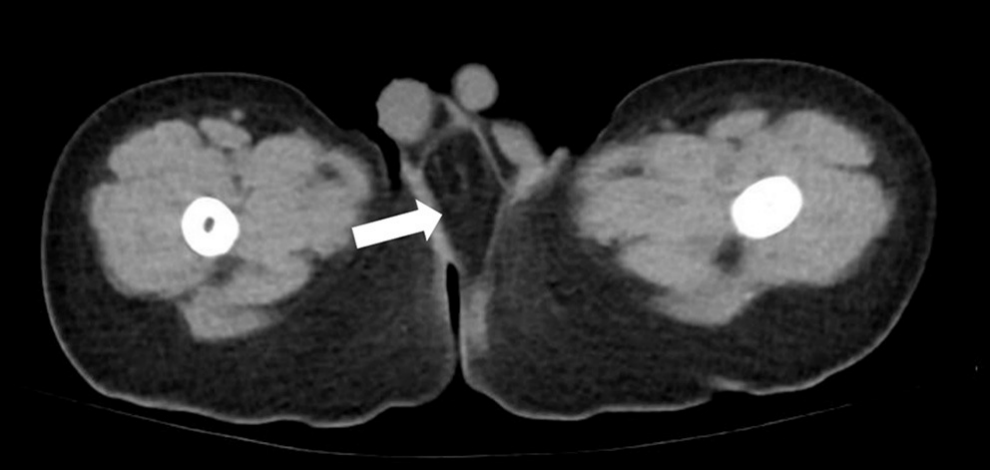
Axial scan on CT showed a low-density mass (white arrow) that had a clear margin at the perineum behind the scrotum (case 7).

ARM associated with labioscrotal fold malformation and perineal mass was diagnosed in seven patients during the past decade in our hospital. Among the patients were six females and one male, and the age at surgery was between 5.2 and 12.4 months. Ultrasound confirmed the existence of normal uterus and ovaries in the females and normal testis in the male patients. Labioscrotal fold malformation appeared to be accessory labioscrotal fold (ALF) in females and accessory scrotum (AS) in the male, with wrinkled scrotal-like skin. The perineal mass was always right behind the labioscrotal fold (Figures 1A–C). Site of the ALF/AS at lateral and mid-perineum was 5:2. The ARM type was all rectoperineal fistula. In addition, one girl had atrial septal defect, and one boy had hypospadia and incomplete caudal duplication syndrome. The other five patients had no other associated anomalies. Ultrasound of the patients showed local mass limited to the subcutaneous layer, without infiltration or connection to the surrounding tissue. For better assessment, the male patient (case 7) underwent a CT scan before surgery, which showed a low-density lesion with clear margins (Figure 2) restricted to the local tissue.

**Figure 3:**
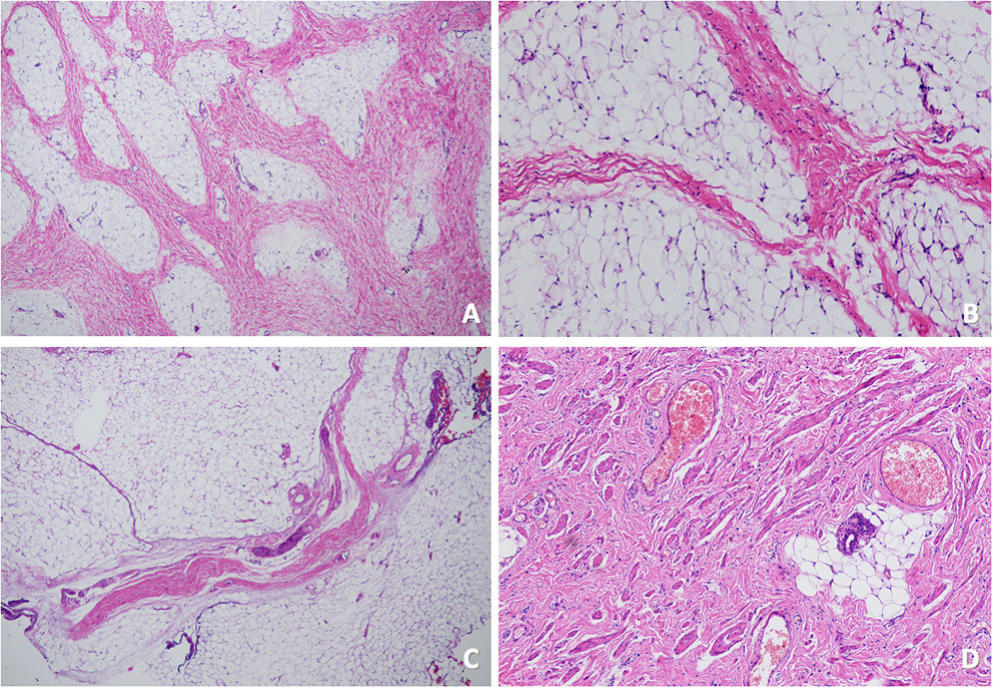
The pathology of case 6 **(A,B)** and case 7 **(C,D)**. **(A)** (HE × 40) and **(B)** (HE × 100) showed the mass consisted of fat and mature fiber tissue. **(C)** (HE × 40) showed the mass mainly consisted of fat and few mature fiber tissues, and **(D)** (HE × 100) showed hyperplasia of smooth muscle bundles and a few skin appendages in the lesion.

Surgery for excision of the ALF/AS and perineal mass occurred simultaneously with anoplasty. Since all seven patients presented with rectoperineal fistula, the anterior perineal approach (Figures 1A–D) was elected, and patients were placed in the lithotomy position after anesthesia. Firstly, we labeled the contraction center of the external anal sphincter as the location for the future anus with the help of bipolar electrical stimulator. Secondly, dissociation close to ALF/AS and mass was accomplished using needle-like electrotome; all seven patients had mass only involving the subcutaneous tissue. Sutures were used for anatomical reduction. Thirdly, anoplasty was done by incising the posterior edge of the fistula to the previously labeled sphincter center, allowing the posterior margin of the anus to be surrounded by external anal sphincter. For the male patient (case 7), he had two external sphincter contraction centers, one was right behind the fistula and the other was on the midline. We chose the contraction center nearer the fistula as his future anus (Figures 1C,D). Pathology was lipoma (three cases), fibroma (one case), lipofibroma (one case; Figures 3A,B), angiolipoma (one case), and mesenchymal hamartoma (one case; Figures 3C,D).

The patients had no wound infection or dehiscence, and during the follow-up period of 7–100 months post-surgery, the median was 63 months, none of the patients suffered perineal mass recurrence. They underwent anal dilation once a day for 6 months, starting from the second week after anoplasty. Bowel control was satisfactory in the follow-up period.

## Discussion

A few studies discussing the three separate anomalies have been reported before (2–5); however, the coexistence of ARM, labioscrotal malformation, and perineal mass in a child is rarely documented in the literature. Pathogenesis, clinical characteristics, treatment, and prognosis may differ from the separate anomaly cases. To date, this is the first consecutive case study concerning the special triad.

Results showed that females might be more prone to this disease when compared with males; however, our study only had a case load of seven, which makes the conclusion susceptible to bias. More cases are required for further analysis. ALF/AS can be divided into two types, lateral type and mid-perineum type (6), and results in this study showed a 5:2 ratio between them. All the reported male patients, three patients from literature (7–9) and one from this study, had mid-perineum-type AS. However, females had a high tendency for lateral-type ALF. Site of the labioscrotal fold malformation is mainly dependent on the interruption of normal development. Development of the scrotum and descending of the testis may affect the formation. In addition, pathology of the perineal mass all turned out to be benign tumor derived from mesenchymal tissue, and type of ARM were all classified as “low” defect without urogenital system anomalies in this study.

Thus, it could be speculated that this triad of ARM, labioscrotal fold malformation, and perineal mass resulted from a caudal developmental disorder. From literature review and current embryological theories (2, 6–8), males and females share the same developing process before the ninth week of embryo. In week 7, with the development of the urorectal septum, the cloacal membrane is divided into the urogenital membrane (anterior) and anal membrane (posterior). Labioscrotal swelling gradually grows, appearing as the labioscrotal fold, surrounding the urogenital membrane and genital fold. From week 9, in females, the labioscrotal fold begins to extend and fuse bidirectionally, which finally forms the posterior labial commissure, anterior mons pubis, and labium major; in males, it grows backward and descends caudally, before fusing posteriorly to form the scrotum. At the same time, tissue derived from the urorectal septum forms the perineum, and with its extension, the anus moves backward and departs from the anterior urogenital system (7). Pathogenesis of the triad can be explained as excessive growing of intervening mesenchymal tissue, which grows to become a perineal mass (8, 10), in turn disrupting the continuity of caudal development of normal labioscrotal fold, and thereby forms the ALF/AS (6, 9); the perineal mass affects the extension of urorectal septum at the perineum (4), leading to a rectoperineal fistula. Aside from ARM in the form of a rectoperineal fistula, previous reports also described rectovestibular fistula and “high” defect of ARM as rectourethra fistula or even cloaca (7, 11, 12) with perineal lipoma but without labioscrotal fold malformation. It is plausible that they may share the same pathogenesis.

Another characteristic is that we found female patients had ALF presenting with wrinkled and pigmented scrotal-like skin (Figure 1A). However, different from females, in the male patient, AS appeared as an excrescence covered by scrotal-like skin bulging on the scrotal fold (Figure 1C). In females, ALF with perineal mass should be distinguished from a Nuck cyst, as they all tend to be found on the labia majora. However, ALF has a particular distinctive skin and the mass has clear margins, which is different from the Nuck cyst that has a changeable volume caused by a congenital patent processus vaginalis. In males, scrotal fold malformation is rare and has a low incidence and can be divided into four types: bifid scrotum, penoscrotal transposition, ectopic scrotum, and accessory scrotum (7, 8, 13, 14). The nature of the four types is failure to fuse or abnormal caudal development of the labioscrotal fold and should be identified from each other.

Post-surgery fecal continence is mainly dependent on the type of ARM. Labioscrotal fold malformation and perineal mass did not increase the complication when they were located subcutaneously without the involvement of the external anal sphincter. In our study, all seven patients had low type of ARM, rectoperineal fistula with mass beyond the anal sphincter, so bowel control was achieved and fecal continence was satisfactory. The male patient in this study had incomplete caudal duplication syndrome. With the use of bipolar electric stimulator during surgery, we noticed two concentration centers on the perineum, one in the midline and the other behind the fistula on the right of the buttock. He had no other caudal anomalies, and in order to minimize operative strike, we chose a posterior incision of the fistula to the nearer center on the right close to the fistula. AS, perineal mass, ARM, and incomplete caudal duplication may all be induced by abnormal caudal development.

In conclusion, ARM associated with labioscrotal fold malformation and perineal mass has a low incidence and is rarely encountered in clinical practice. It may have a female prevalence. Nature of the triad is the neoplastic overgrowth of intervening mesenchymal tissue, impeding the continuity of caudal development of normal labioscrotal fold, affecting the extension of the urorectal septum and leading to ARM. Prognosis is mainly dependent on the type of ARM.

## Data Availability Statement

The original contributions presented in the study are included in the article/supplementary material, further inquiries can be directed to the corresponding author/s.

## Ethics Statement

The studies involving human participants were reviewed and approved by Medical Ethics Committee of Beijing Children's Hospital, Capital Medical University (2020-Z-127). Written informed consent from the participants' legal guardian/next of kin was not required to participate in this study in accordance with the national legislation and the institutional requirements.

## Author Contributions

KW and YC contributed to the study conception and design. CP, WP, ZW, DW, and DZ contributed to data acquisition. KW and SS contributed to the analysis and data interpretation. KW contributed to the drafting of the manuscript. YC and SS contributed to the critical revision. All authors contributed to the article and approved the submitted version.

## Conflict of Interest

The authors declare that the research was conducted in the absence of any commercial or financial relationships that could be construed as a potential conflict of interest.
